# Supplementary material for: Comprehensive identification of mRNA isoforms reveals the diversity of neural cell-surface molecules with roles in retinal development and disease
Source: Nat Commun. 2020 Jul 3;11:3328. doi: 10.1038/s41467-020-17009-7 (PMC7335077; doi:10.1038/s41467-020-17009-7)
Supplement: Supplementary file 1 — Supplementary Information [file 41467_2020_17009_MOESM1_ESM.pdf]

SUPPLEMENTARY INFORMATION

Comprehensive identification of mRNA isoforms reveals the diversity of neural cell-surface molecules with roles in retinal development and disease

Thomas A. Ray et al.

Table of contents:

1. Supplementary Figures 1-7

2. Supplementary Tables 1-2

3. Supplementary References

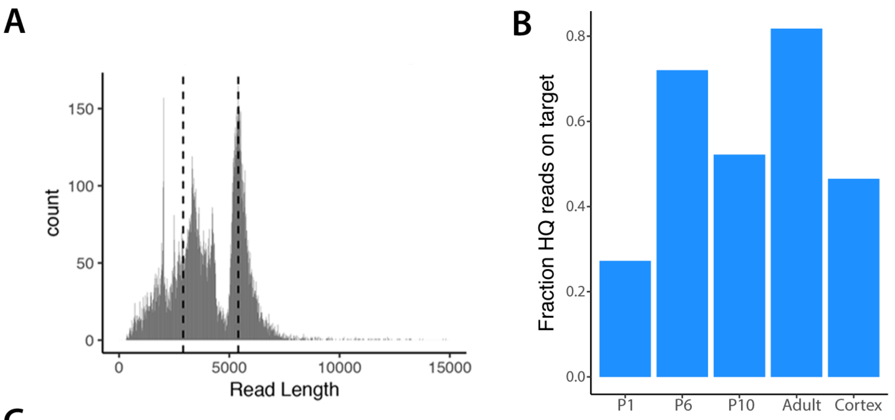

C

Sequencing Profiles						
	P1 Retina	P6 Retina	P10 Retina	Adult Retina	Adult Cortex	Combined
Number of SMRT Cells	9	8	8	1 Sequel	2 Sequel	25 SMRT/3 Sequel
Reads of Insert	255,930	362,892	397,192	236,025	71,116	1,323,155
Number of 5 Prime Reads	130,167	191,980	225,931	198,831	62,689	809,598
Number of Poly-A Reads	153,200	206,252	246,590	209,625	66,797	882,464
Number of Non-Full-Length Reads	153,214	179,274	207,406	59,504	9,941	609,339
Number of Full-Length Non-Chimeric Reads	89,675	157,898	179,414	176,457	61,159	664,603
Average Full-Length Non-Chimerc Read Length (bp)	5,515	4,427	5,063	4,576	4,937	4,832

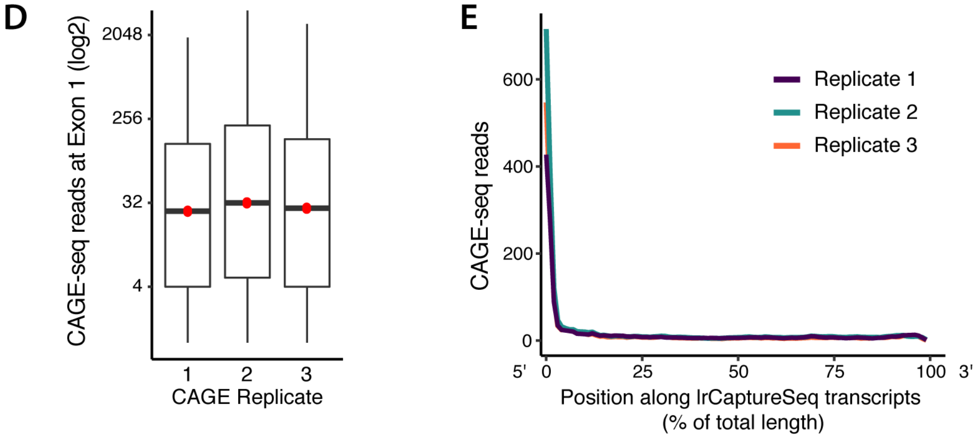

Supplementary Figure 1. PacBio sequencing of captured cDNAs

(**A**) Histogram of PacBio read size distribution for a pilot lrCaptureSeq experiment, in which the second size selection after PCR amplification was not performed (see workflow, Fig. 1B. Profile demonstrates that this size selection is necessary for enrichment of long transcripts. Dotted line represents interquartile range. FLNC, full-length non-chimeric reads called by IsoSeq software.

(**B**) Percentage of on target reads per experiment, calculated as the number of high quality (HQ) reads corresponding to our targeted genes vs. all other reads. HQ reads called by IsoSeq software.

(**C**) Sequencing statistics from each individual lrCaptureSeq experiment and the combined dataset.

(**D,E**) Validation of lrCaptureSeq isoform 5' ends by CAGE. Three independent CAGE-seq replicates from adult mouse retina were mapped to the adult mouse retina lrCaptureSeq isoforms. D: Box and whiskers plot showing CAGE read coverage at the first exon of lrCaptureSeq isoforms (n = 1076). Coverage is extensive, supporting the accuracy of lrCaptureSeq 5' ends. Box represents IQR, horizontal line represents median, whiskers equal to 1.5*IQR. E: Position along 5'-3' axis of CAGE reads that mapped to lrCaptureSeq isoforms. CAGE coverage was exclusive to 5' end of transcripts.

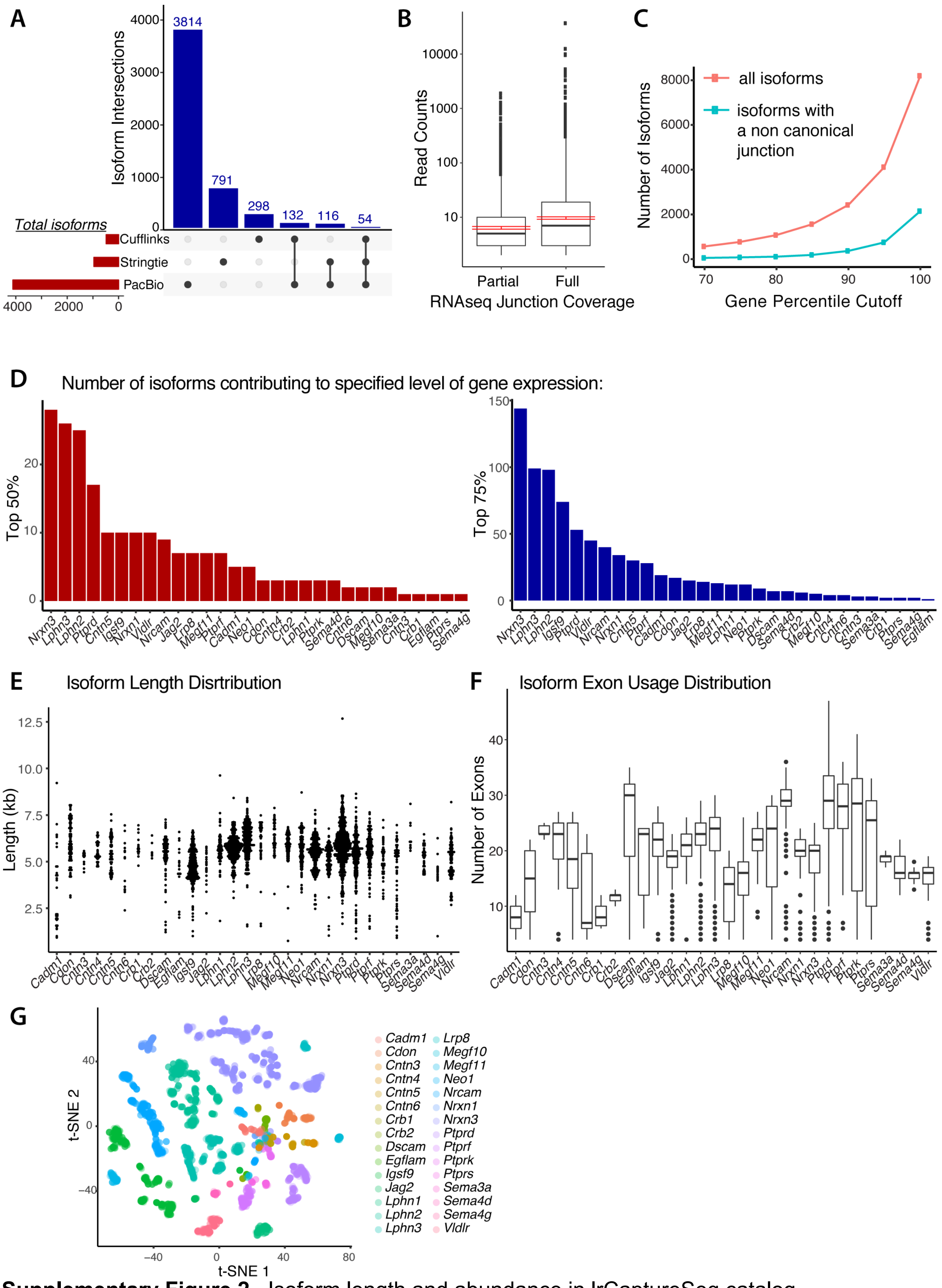

Supplementary Figure 2. Isoform length and abundance in IrCaptureSeq catalog

(**A**) UpSet plot comparing number of “ground-truth” isoforms in the IrCaptureSeq dataset with ones

computationally predicted from retina and cortex RNA-seq datasets by Cufflinks or Stringtie. Many more isoforms were detected by IrCaptureSeq than were assembled by these two programs. Nevertheless, only a minority of predicted isoforms were validated by long-read sequencing: 186 isoforms predicted by Cufflinks (3rd + 5th columns) were detected in the PacBio dataset (or 38% of Cufflinks isoforms), and 170 isoforms predicted by Stringtie (4th + 5th columns) were detected (or 17.7% of Stringtie isoforms).

(**B**) Box and whisker plot showing number of RNA-seq reads that mapped to IrCaptureSeq isoforms. Two classes of isoforms are compared: those for which all exon junctions were validated in RNA-seq data (Full; *n* = 2,925), and those that were not 100% validated (Partial; *n* = 1,191). Read counts were lower for the latter group, suggesting that failure to validate all junctions may have resulted at least in part from low expression levels and/or insufficient RNA-seq read coverage of those particular isoforms. Box represents IQR, horizontal line represents median, whiskers equal to 1.5*IQR. Red bar indicates 95% confidence interval of the mean.

(**C**) Contribution of isoforms containing non-canonical splice junctions to overall isoform count. Curves show abundance rank ordering of all isoforms (red), and the same rank ordering for only those isoforms that contain a non-canonical splice junction (blue). Non-canonical junctions account for a small fraction of total isoforms. Successively removing the least abundant isoforms from each gene (i.e. moving along the X axis) yields a similar fraction of isoforms that use a non-canonical junction, suggesting some of these are abundantly expressed.

(**D**) Plots depicting the number of isoforms that account for the top 50% (D) or 75% (E) of each gene's total read count (see Fig. 2C). These plots show that, even with strict abundance cutoffs, many isoforms exist and contribute to overall gene expression.

(E,F) Isoforms vary substantially in their length. This is shown by a dotplot depicting the lengths of isoforms for each gene (F) and by a box and whiskers plot depicting the number of exons used across isoforms of each gene. Box represents IQR, horizontal line represents median, whiskers equal to 1.5*IQR. Sample sizes: The number of isoforms plotted for each gene is given in Fig. 2A.

(**G**) t-SNE plot of all isoforms. Most isoforms segregate into their respective gene families, validating efficacy of clustering algorithms for comparing isoform similarity. Isoforms in center of plot that do not segregate well generally contain large genomic elements (i.e. retained introns) which impede clustering with other isoforms of the same gene. The spread of isoforms suggests significant variations in sequence composition. Plot was generated with 1,000 iterations and perplexity = 35.

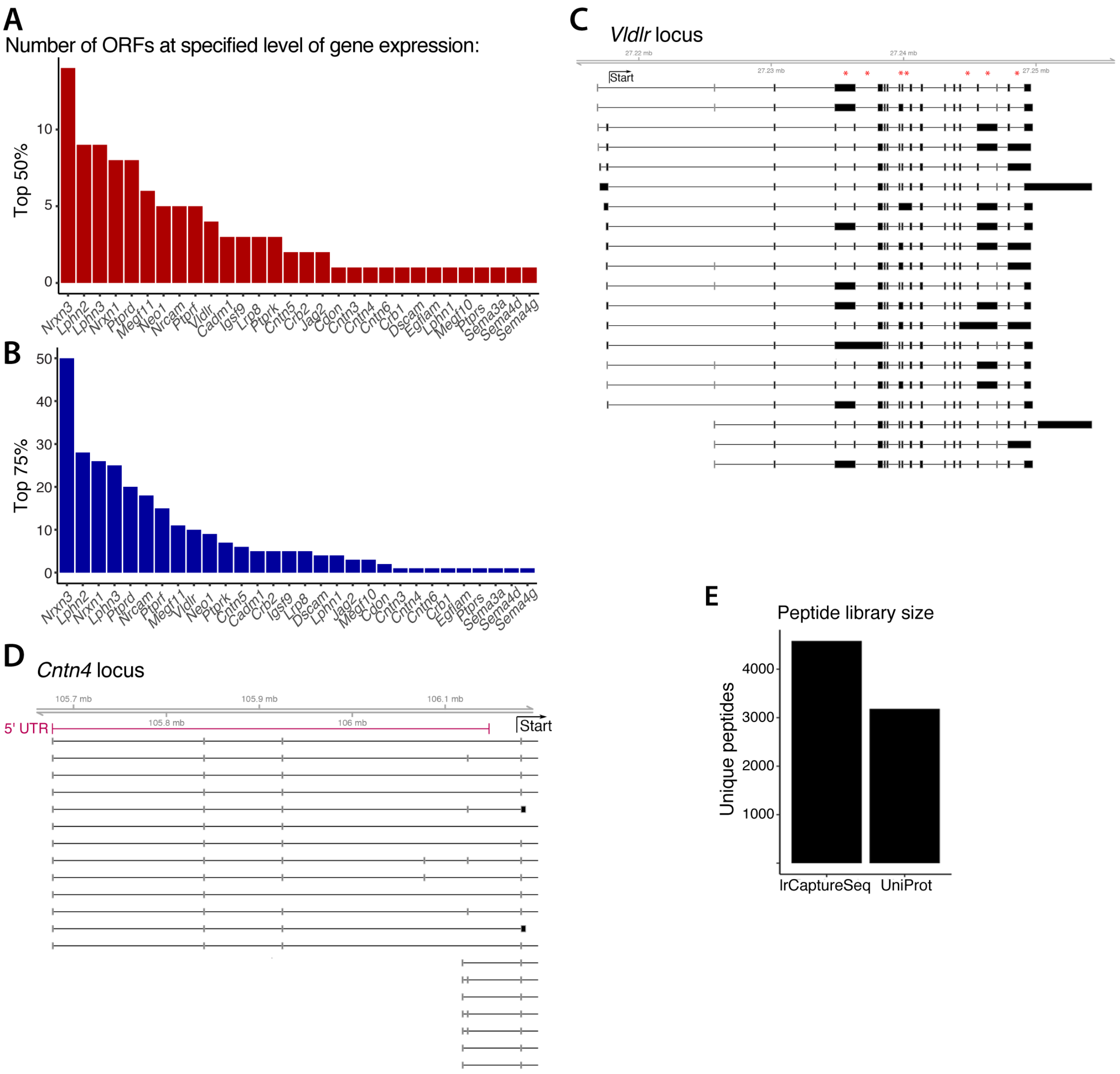

Supplementary Figure 3. Coding and non-coding isoform variations

(A,B) Plots depicting the number of unique predicted ORFs that account for the top 50% (A) or 75% (B) of each gene's total read count (see Fig. 3B).

(C) Intron retention is a major source of non-protein-coding isoform diversity, as exemplified here by *Vldlr* gene. The top 20 most abundant *Vldlr* isoforms are illustrated. Thick black bars, exons. Note extensive, combinatorial intron retention. Asterisks, introns that were detected in IrCaptureSeq isoforms (i.e. within polyadenylated transcripts). Intron retention creates a high degree of transcript diversity that does not translate to high ORF diversity. All of the retained introns introduce premature stop codons.

(D) Non-coding transcript diversity can arise from variations in the 5' UTR region of the gene, as exemplified here by *Cntn4*. Figure shows 5' end of top 20 most abundant *Cntn4* isoforms. Note alternative transcriptional start sites and differential exon usage within 5' UTR.

(E) The number of unique trypsin peptide products encoded by our 30 genes in the UniProtKb database (right bar), compared to the number of predicted trypsin peptide products that exist within the IrCaptureSeq dataset (left bar).

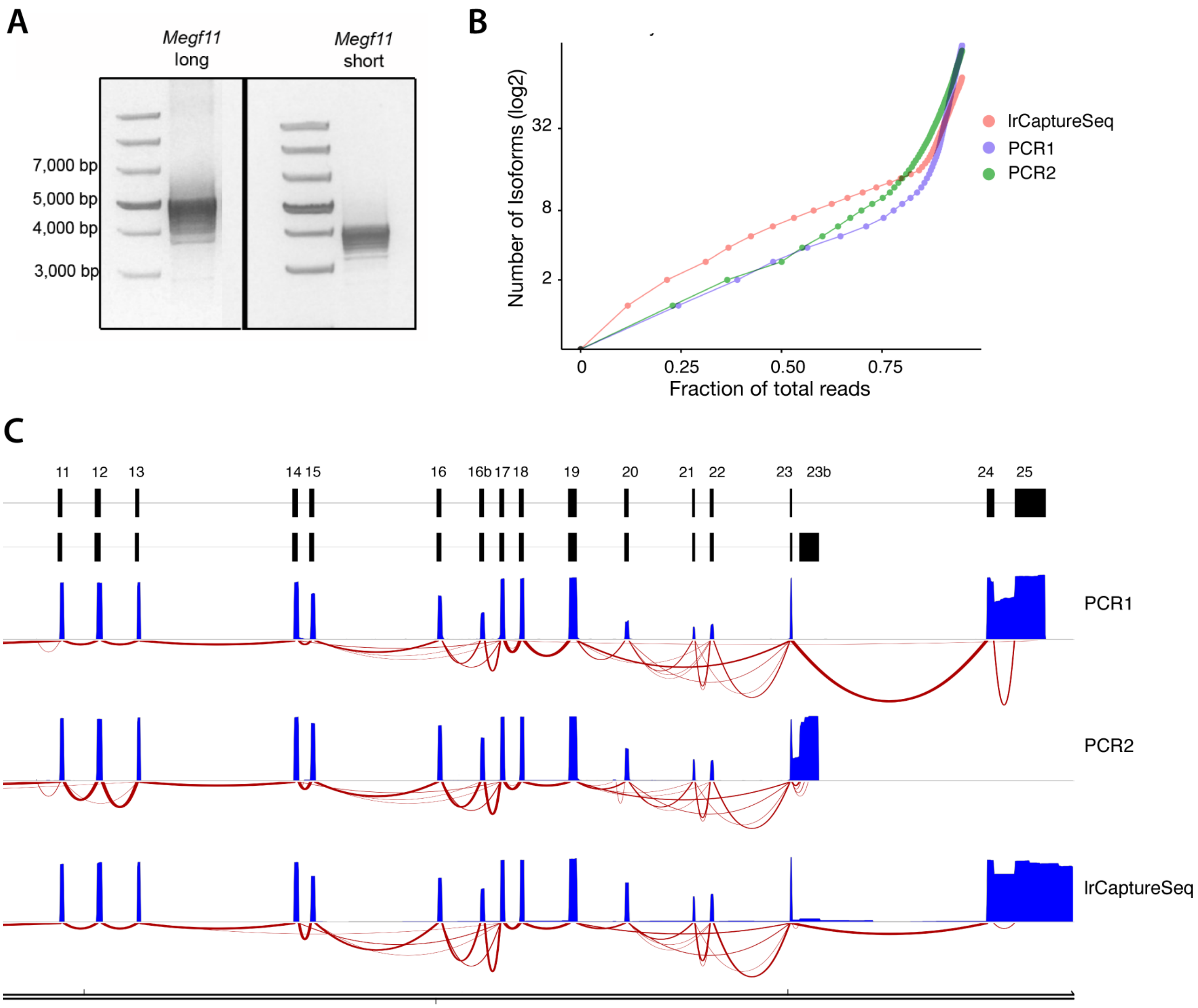

Supplementary Figure 4. *Megf11* isoform diversity uncovered by PacBio sequencing

(A) Representative DNA electrophoresis gel image of *Megf11* RT-PCR products. Primers were designed to amplify two different *Megf11* variants (denoted long and short) by placing primers in exon 25 or alternative exon 23, respectively. PCR was performed on retinal (long) or cortex (short) cDNA. The size spread of RT-PCR products indicates that numerous *Megf11* isoforms of different sizes can be readily amplified.

(B) Lorenz plot profiles of *Megf11* isoform abundance from lrCaptureSeq and PCR datasets. All datasets suggest that many isoforms contribute to overall *Megf11* expression. The rightward shift of the PCR dataset curves suggests overrepresentation of the most abundant isoforms, likely due to PCR-induced bias.

(C) Transcript maps depicting the long and short forms of *Megf11* (top) and corresponding exon coverage (blue) and sashimi plots (red) from 3 different PacBio sequencing datasets. PCR1 dataset was generated by sequencing *Megf11* long form PCR products, while PCR2 dataset was generated by sequencing short form PCR products. These are compared to the *Megf11* reads from the 30-gene lrCaptureSeq experiment. All three experiments reveal extensive alternative splicing of Megf11 transcripts. Sashimi plots show remarkable similarity between the different datasets.

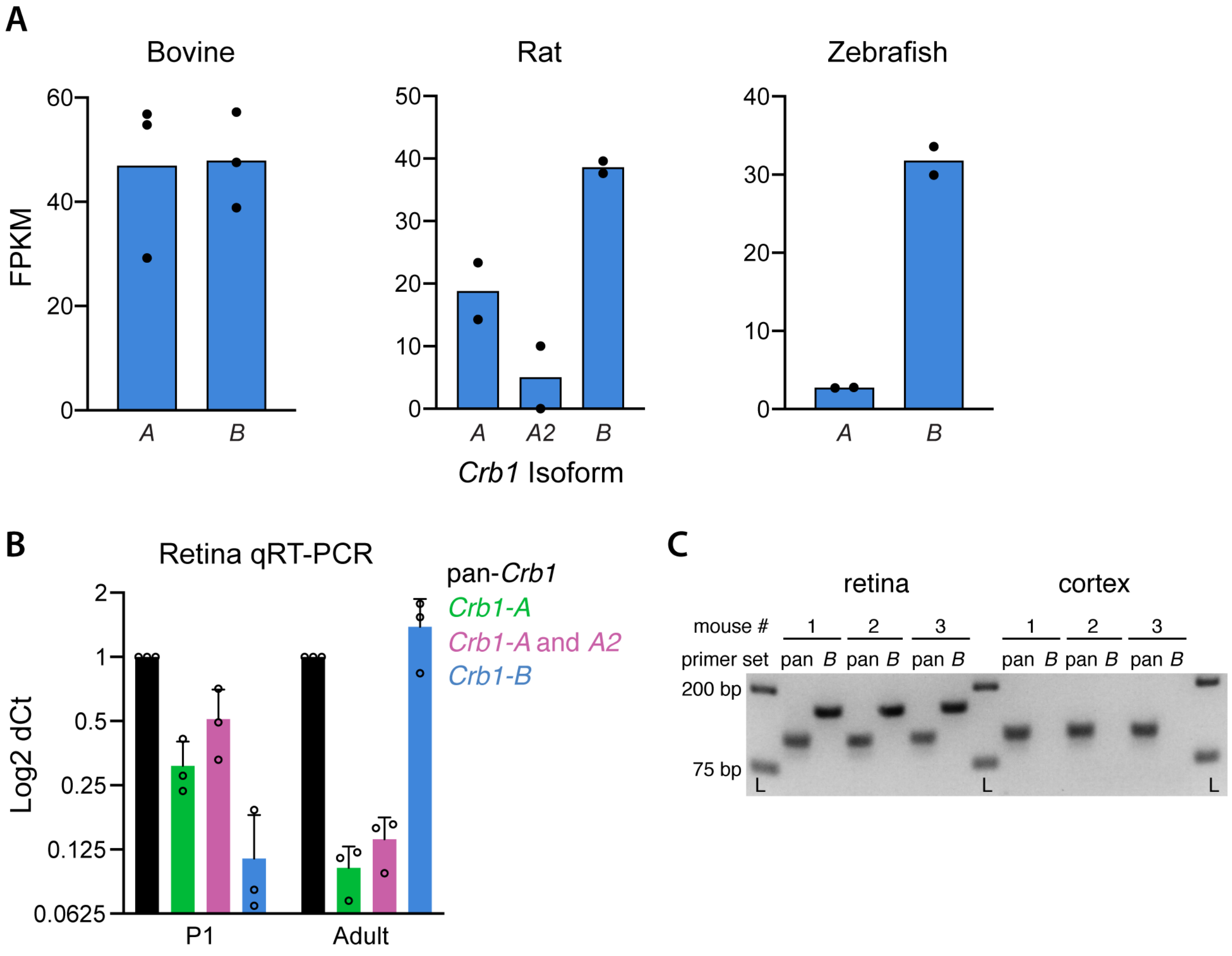

<u>**Supplementary Figure 5.** *Crb1-B* isoform is expressed across a variety of vertebrate species</u>

(A) Quantification of *Crb1* isoforms in bovine, rat, and zebrafish retina, based on publicly available RNA-seq data (bovine, GSE59911; rat, GSE84932; zebrafish, GSE101544). *Crb1-B* is at least as abundant as *Crb1-A* in all species, and is more abundant in rat and zebrafish. *Crb1-A2* was not detectable in bovine or zebrafish retina. Sample sizes (biological replicates): bovine, n = 3; rat, n = 2; zebrafish n = 2.

(B) Quantitative (q) RT-PCR analysis of *Crb1* isoforms in mouse retina confirm expression patterns identified using PacBio and short-read RNA-seq (Fig. 5). *Crb1-A* is most abundant at P1, while *Crb1-B* is most abundant in adulthood. PCR primers were designed to span splice junctions expressed by the indicated isoforms. Data were normalized to values obtained from pan-*Crb1* primers. Sample sizes: n = 3 animals for each age. Error bars, S. E. M.

(C) Representative gel image showing RT-PCR on cDNA from mouse retina and cortex, using pan-*Crb1* primers (*pan*), or primers targeting a *Crb1-B* splice junction (*B*). No *Crb1-B* band is detected in mouse cortex. Pan-*Crb1* primers produce bands in both tissues. N = 3 mice. L, ladder.

See Source Data file for values underlying graphs (A,B) and full gel (C).

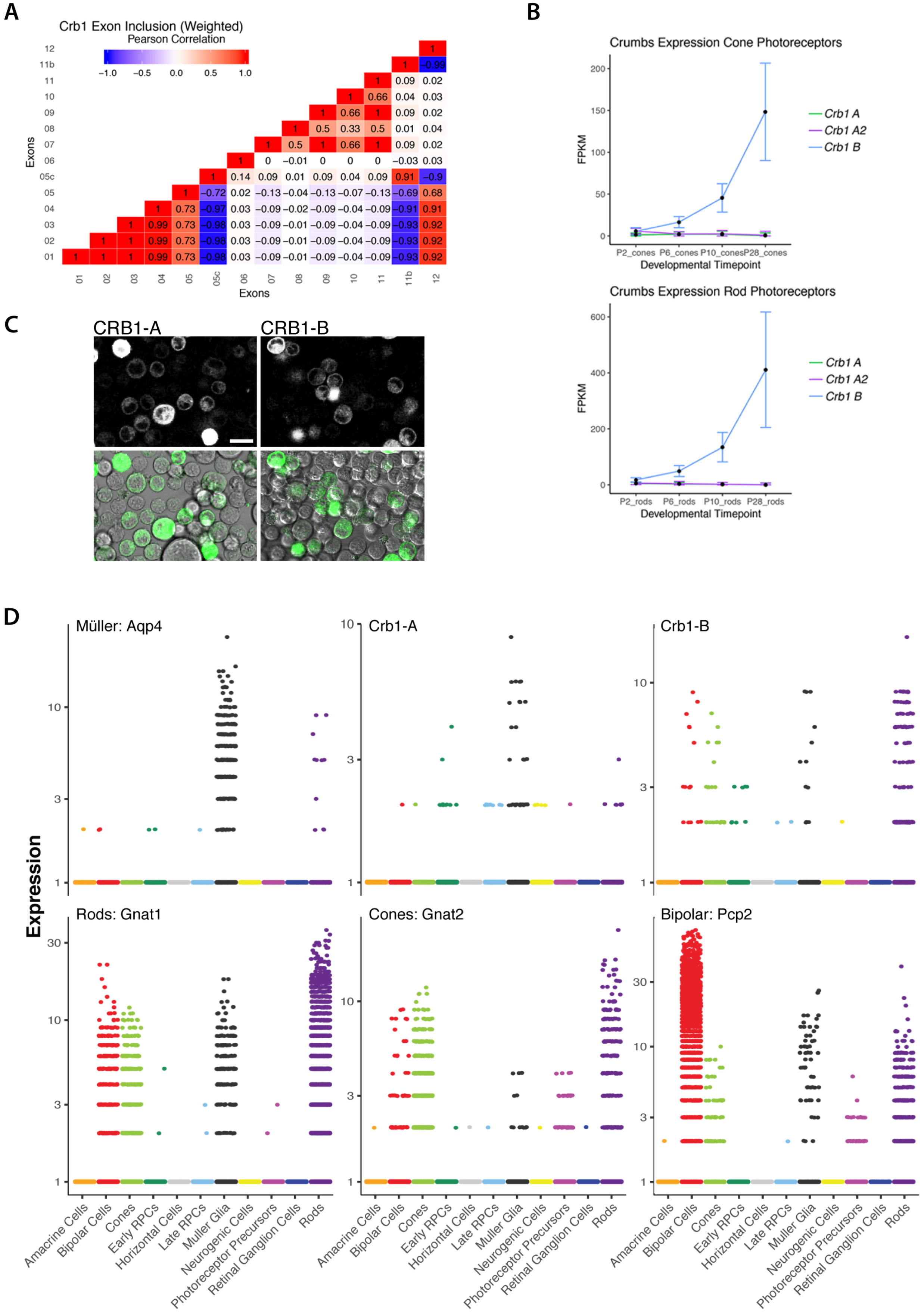

Supplementary Figure 6. Cell-type-specific expression of *Crb1* isoforms

(A) Pearson correlation of *Crb1* exons demonstrates that exons unique to *Crb1-B* (5c and 11b) are negatively correlated with exons unique to *Crb1-A* isoforms (1-5 and 12). The unique *Crb1-B* exons (5c and 11b) are strongly positively correlated suggesting that they are primarily used together.

(B) Quantification of *Crb1* isoforms from bulk RNA-seq of isolated cone (top) and rod (bottom) photoreceptors (dataset: GSE74660). *Crb1-B* is the only isoform expressed in photoreceptors. Sample sizes (biological replicates): P2 rods and cones, $n = 2$; P6 rods and cones, $n = 3$; P10 rods and cones, $n = 3$; P28 rods, $n = 4$; P28 cones, $n = 2$. For values see Source Data file. Error bars, 95% confidence intervals of the FPKM value computed by Cufflinks software.

(C) CRB1 isoforms expressed in K562 cells traffic to the plasma membrane. Images depict native fluorescence of CRB1-A and CRB1-B constructs tagged at C-terminus with YFP. Scale bar, 25 µm.

(D) Mapping of *Crb1* isoforms in single-cell RNA-seq data. Jitter plot indicates relative transcript expression counts within individual cells. Each point represents one cell, colored by the annotated cell type. *Crb1-A* is expressed by Müller glia whereas *Crb1-B* is expressed by rod and cone photoreceptors. Cell type-specific markers of Müller glia (*Aqp4)*, rods (*Gnat1*), cones (*Gnat2*), and bipolar cells (*Pcp2*) are shown for comparison.

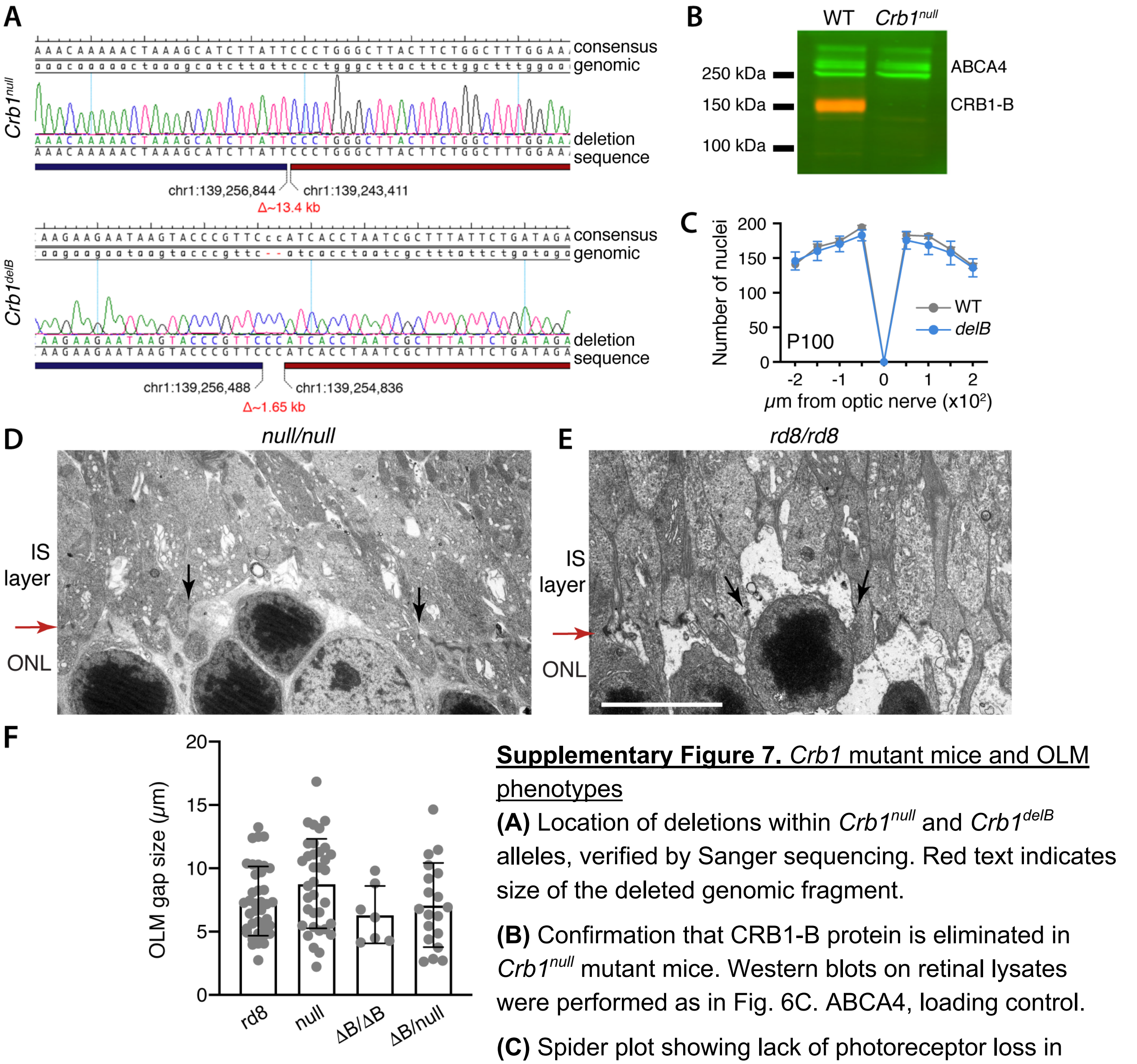

Supplementary Figure 7. *Crb1* mutant mice and OLM phenotypes

(A) Location of deletions within *Crb1*^{null} and *Crb1*^{delB} alleles, verified by Sanger sequencing. Red text indicates size of the deleted genomic fragment.

(B) Confirmation that CRB1-B protein is eliminated in *Crb1*^{null} mutant mice. Western blots on retinal lysates were performed as in Fig. 6C. ABCA4, loading control.

(C) Spider plot showing lack of photoreceptor loss in *Crb1*$^{delB/delB}$ mice at P100. Gray, wild-type controls. For sample sizes see legend to Fig. 10c.

(D,E) Representative electron micrographs showing OLM disruptions in *Crb1*^{null} and *Crb1*^{rd8} mutants. Images are similar in scale to Fig. 7D,E. Arrows demarcate region lacking OLM junctions. Anatomical disturbances are similar to those previously reported for *rd8*[36], and to those observed in *Crb1*$^{delB/null}$ mice, which lack *Crb1-B* but still retain one copy of *Crb1-A* (Fig. 7). Scale bar, 5 μm.

(F) OLM gap size in *Crb1* mutants carrying various allele combinations. Size of OLM gaps was not significantly different across the various mutants. Statistics, one-way ANOVA. Main effect of genotype: $F(3, 95) = 2.19$; $P = 0.095$).

Also see Source Data file (panels B,C,F). Sample sizes: *Rd8*, *n* = 38 gaps from 3 mutants; *null*, *n* = 35 gaps from 3 mutants; *delB/delB* *n* = 7 gaps from 3 mutants; *delB/null*, *n* = 19 gaps from 5 mutants. Error bars, S. E. M.

SUPPLEMENTARY TABLES

	chr12 coordinates:		Isoforms:		Total reads:	
Nrxn3 Exon	Start	Stop	n	Frequency	n	Frequency
1.1 TSS	88722853	88723186	326	0.37	16281	0.64
1	88795185	88795893	634	0.71	21483	0.84
**AS1**						
2	88832553	88832570	639	0.72	20576	0.81
3	88850279	88850308	621	0.70	20325	0.80
4	88853105	88853116	76	0.09	1542	0.06
**AS2**						
6.1	89191153	89191197	19	0.02	1451	0.06
6.2	89191153	89191176	150	0.17	2654	0.10
**AS3**						
10	89348246	89348449	782	0.88	20533	0.81
11	89354453	89354479	611	0.69	16340	0.64
11.1 TTS	89387021	89391046	3	0.00	30	0.00
17.3 TTS	89577190	89578637	2	0.00	8	0.00
17.1 TTS	89651669	89653007	3	0.00	25	0.00
17.2 TTS	89651903	89653007	5	0.01	65	0.00
18 Beta	89813400	89813640	13	0.01	1267	0.05
19.1 TTS	90091254	90092125	1	0.00	4	0.00
**AS4**						
20	90168920	90169091	717	0.80	19955	0.78
21	90199147	90199236	420	0.47	12252	0.48
21.1 TTS	90201006	90201668	1	0.00	7	0.00
22.1 TTS	90214224	90215613	1	0.00	5	0.00
**AS5**						
24.1	90321936	90322641	122	0.14	2453	0.10
24.2	90322285	90322641	0	0.00	0	0.00
24.3	90322460	90322641	0	0.00	0	0.00
24.4	90322539	90322641	0	0.00	0	0.00
25.1	90331485	90332410	233	0.26	6014	0.24
25.2	90331788	90332410	221	0.25	5605	0.22
25.3	90332109	90332410	221	0.25	5927	0.23

Supplementary Table 1: Exon use at *Nrxn3* alternative splice sites.

To assess efficiency of lrCaptureSeq, well-characterized splice sites within the *Nrxn3* gene were analyzed. This table gives the frequency with which exons in each alternative splice (AS) site was detected, per isoform and per total *Nrxn3* reads. Blue shading indicates frequency of exon use: low (light blue) to high (dark blue). Yellow, new exons identified by lrCaptureSeq.

Supplementary Table 2: Key Resources

Reagent Type	Reagent	Source or reference	Identifier	Additional information
Antibody	Alexa Fluor 488 AffiniPure Donkey Anti-rabbit IgG: 1:1000	Jackson ImmunoResearch	711-545-152	
Antibody	Rabbit anti-Calbindin 1:5000	Swant	CB-38	
Antibody	Rabbit anti-CRB1-B: 1:500	this study	–	
Antibody	goat anti-ABCA4 1:1000	Santa Cruz	SC21460	
Antibody	mouse anti rhodopsin clone 1D4 1:1000	Abcam	ab5417	
Antibody	mouse anti-GAPDH clone 0411 1:1000	Santa Cruz	sc-47724	
Antibody	Sheep anti-phosducin 1:5000	Ref [1]	–	
Antibody	IRDye 800CW Donkey anti-Rabbit IgG (H + L): 1:1000	Li-Cor Biosciences	925–32213	
Antibody	IRDye 680RD Donkey anti-Mouse IgG (H + L): 1:1000	Li-Cor Biosciences	925–68072	
biological reagent	KAPA HiFi DNA Polymerase	Kapa Biosystems	KK2602	
biological reagent	Takara LA Taq	Takara Bio	RR002A	
biological reagent	Nimblegen's SeqCap EZ Developer (≤200Mb) custom baits	Nimblegen	–	Capture probes
biological reagent	Twist Bioscience NGS Taret Enrichment	Twist Bioscience	–	Capture probes
biological reagent	Phusion High-Fidelity DNA Polymerase	New England Biolabs	M0530S	
biological reagent	Trypsin/Lys-C Mix, Mass Spec Grade	Promega	v072	
Chemical compound	16% Paraformaldehyde	Electron Microscopy Sciences	15710	
Chemical compound	50% Glutaraldehyde	Electron Microscopy Sciences	G5882	

Supplementary Table 2: Key Resources

Reagent Type	Reagent	Source or reference	Identifier	Additional information
Chemical compound	Normal Donkey Serum	Jackson ImmunoResearch	017-000-121	
Chemical compound	TriReagent	Thermo Fisher Scientific	AM9738	
Chemical compound	Hank's balanced salt solution (HBSS)	Sgma Aldrich	H8264	
Chemical compound	Fetal Bovine Serum	Life Technologies	16250–078	
Chemical compound	Reduced Serum Medium	Thermo Fisher Scientific	31985070	
Chemical compound	Vecta-Mount	Vector Laboraories	H-5000	
Chemical compound	ammonium bicarbonate	Sigma-Aldrich	11213	
Chemical compound	Iodoacetamide (IAA)	Sigma-Aldrich	I1149	
Chemical compound	Dithiothreitol (DTT)	Sigma-Aldrich	43815	
Chemical compound	Pierce™ Streptavidin Magnetic Beads	Thermo Fischer Scientific	88816	
Chemical compound	EZ-Link™ Sulfo-NHS-SS-Biotin	Thermo Fischer Scientific	21328	
Chemical compound	Hoechst 33258	Invitrogen	H21491	
Chemical compound	Isothesia: Isoflurane	Henry Schein	11695–6776	
Chemical compound	Tissue Freezing Medium	VWR	15148–031	
Chemical compound	4x Laemmli Sample Buffer	Bio-Rad	1610747	
Chemical compound	Odyssey Blocking Buffer	Li-Cor Biosciences	927–40000	
Chemical compound	EDTA-free Protease Inhibitor Cocktail Tablets	Roche	4693159001	
Other	Immun-Blot Low Fluorescence PVDF membrane	Bio-Rad	1620264	
commercial assay or kit	Bio-Rad DC Protein Assay Kit	Bio-Rad	5000112	
recombinant DNA	CAG-Crb1-B-YFP	this study		
recombinant DNA	CAG-Crb1-A-YFP	this study		

Supplementary Table 2: Key Resources

Reagent Type	Reagent	Source or reference	Identifier	Additional information
recombinant DNA	CAG-YFP	Addgene	11180	
Cell line	K562	ATCC	CCL-243	
model organism	Mouse: C57Bl6/J	Jackson Labs	000664	
model organism	Mouse: CD-1	Charles River	022	
model organism	Mouse: Crb1null	this study		
model organism	Mouse: Crb1delB	this study		
model organism	Mouse: B6SJLF1/J	Jackson Labs	100012	
model organism	Mouse: C57Bl6/N	Charles River	027	
Software	Prism	Graphpad		
Software	Fiji/ImageJ	Ref [2]		
Software	Cufflinks	Ref [3]		
Software	CummeRbund	Ref [3]		
Software	StringTie	Ref [4]		
Software	Hisat2	Ref [5]		
Software	SQANTI	Ref [6]		
Software	NIS Elements	Nikon Instruments		
Software	Image StudioTM	LI-COR Biosciences		
Software	Lasergene	DNASTAR		
Software	Photoshop	Adobe		
Software	Integrated Genome Browser (IGV) 2.4.16	Ref [7]		
Software	STAR	Ref [8]		
Software	Gviz	Ref [9]		
Software	Iso-Seq	Pacific Biosciences		
Software	SMART embl	Ref [10]		
Software	text2vec	Ref [11]		used for k-mer counting
Software	treemapify	Ref [12]		

Supplementary Table 2: Key Resources

Reagent Type	Reagent	Source or reference	Identifier	Additional information
Software	UpSetR	Ref [13]		
Software	GMAP	Ref [14]		
Software	R v3.3.3	R-Project		
Software	Tidyverse R packages ggplot2, dplyr, stringr, magrittr	Ref [15]		
Software	reshape2 R package	Ref [16]		used in making correlation heatmaps
Software	plotly R package	Dash Enterprise		used for 3D plots
Software	dendextend R package	Ref [17]		used in clustering dendrogram tree plots
Software	Rtsne R package	Ref [18]		used for t-SNE
Software	vegan R package	Ref [19]		used to calculate Shannon Index
Software	IsoPops	Ref [20]		Analysis and visualization of long-read data. Introduced in this study
GEO dataset GSE74660	P2_rep1	Ref [21]	SRR2936836	mouse photoreceptor bulk RNA-seq (Supplementary Fig. 6B)
GEO dataset GSE74660	P2_rep2	Ref [21]	SRR2936837	mouse photoreceptor bulk RNA-seq (Supplementary Fig. 6B)
GEO dataset GSE74660	P4_rep1	Ref [21]	SRR2936838	mouse photoreceptor bulk RNA-seq (Supplementary Fig. 6B)
GEO dataset GSE74660	P4_rep2	Ref [21]	SRR2936839	mouse photoreceptor bulk RNA-seq (Supplementary Fig. 6B)
GEO dataset GSE74660	P6_rep1	Ref [21]	SRR2936840	mouse photoreceptor bulk RNA-seq (Supplementary Fig. 6B)
GEO dataset GSE74660	P6_rep2	Ref [21]	SRR2936841	mouse photoreceptor bulk RNA-seq (Supplementary Fig. 6B)
GEO dataset GSE74660	P6_rep3	Ref [21]	SRR2936842	mouse photoreceptor bulk RNA-seq (Supplementary Fig. 6B)
GEO dataset GSE74660	P10_rep1	Ref [21]	SRR2936843	mouse photoreceptor bulk RNA-seq (Supplementary Fig. 6B)

Supplementary Table 2: Key Resources

Reagent Type	Reagent	Source or reference	Identifier	Additional information
GEO dataset GSE74660	P10_rep2	Ref [21]	SRR2936844	mouse photoreceptor bulk RNA-seq (Supplementary Fig. 6B)
GEO dataset GSE74660	P10_rep3	Ref [21]	SRR2936845	mouse photoreceptor bulk RNA-seq (Supplementary Fig. 6B)
GEO dataset GSE74660	P14_rep1	Ref [21]	SRR2936846	mouse photoreceptor bulk RNA-seq (Supplementary Fig. 6B)
GEO dataset GSE74660	P14_rep2	Ref [21]	SRR2936847	mouse photoreceptor bulk RNA-seq (Supplementary Fig. 6B)
GEO dataset GSE74660	P28_rep1	Ref [21]	SRR2936848	mouse photoreceptor bulk RNA-seq (Supplementary Fig. 6B)
GEO dataset GSE74660	P28_rep2	Ref [21]	SRR2936849	mouse photoreceptor bulk RNA-seq (Supplementary Fig. 6B)
GEO dataset GSE74660	P28_rep3	Ref [21]	SRR2936850	mouse photoreceptor bulk RNA-seq (Supplementary Fig. 6B)
GEO dataset GSE74660	P28_rep4	Ref [21]	SRR2936851	mouse photoreceptor bulk RNA-seq (Supplementary Fig. 6B)
GEO dataset GSE74660	P2_KO_rep1	Ref [21]	SRR2936852	mouse photoreceptor bulk RNA-seq (Supplementary Fig. 6B)
GEO dataset GSE74660	P2_KO_rep2	Ref [21]	SRR2936853	mouse photoreceptor bulk RNA-seq (Supplementary Fig. 6B)
GEO dataset GSE74660	P4_KO_rep1	Ref [21]	SRR2936854	mouse photoreceptor bulk RNA-seq (Supplementary Fig. 6B)
GEO dataset GSE74660	P4_KO_rep2	Ref [21]	SRR2936855	mouse photoreceptor bulk RNA-seq (Supplementary Fig. 6B)
GEO dataset GSE74660	P6_KO_rep1	Ref [21]	SRR2936856	mouse photoreceptor bulk RNA-seq (Supplementary Fig. 6B)
GEO dataset GSE74660	P6_KO_rep2	Ref [21]	SRR2936857	mouse photoreceptor bulk RNA-seq (Supplementary Fig. 6B)
GEO dataset GSE74660	P6_KO_rep3	Ref [21]	SRR2936858	mouse photoreceptor bulk RNA-seq (Supplementary Fig. 6B)
GEO dataset GSE74660	P10_KO_rep1	Ref [21]	SRR2936859	mouse photoreceptor bulk RNA-seq (Supplementary Fig. 6B)

Supplementary Table 2: Key Resources

Reagent Type	Reagent	Source or reference	Identifier	Additional information
GEO dataset GSE74660	P10_KO_rep2	Ref [21]	SRR2936860	mouse photoreceptor bulk RNA-seq (Supplementary Fig. 6B)
GEO dataset GSE74660	P10_KO_rep3	Ref [21]	SRR2936861	mouse photoreceptor bulk RNA-seq (Supplementary Fig. 6B)
GEO dataset GSE74660	P14_KO_rep1	Ref [21]	SRR2936862	mouse photoreceptor bulk RNA-seq (Supplementary Fig. 6B)
GEO dataset GSE74660	P14_KO_rep2	Ref [21]	SRR2936863	mouse photoreceptor bulk RNA-seq (Supplementary Fig. 6B)
GEO dataset GSE74660	P28_KO_rep1	Ref [21]	SRR2936864	mouse photoreceptor bulk RNA-seq (Supplementary Fig. 6B)
GEO dataset GSE74660	P28_KO_rep2	Ref [21]	SRR2936865	mouse photoreceptor bulk RNA-seq (Supplementary Fig. 6B)
GEO dataset GSE102092	E14.5	Ref [22]	SRR5884802	ATAC-seq (Fig. 5C)
GEO dataset GSE102092	E17.5	Ref [22]	SRR5884803	ATAC-seq (Fig. 5C)
GEO dataset GSE102092	P0	Ref [22]	SRR5884804	ATAC-seq (Fig. 5C)
GEO dataset GSE102092	P3	Ref [22]	SRR5884805	ATAC-seq (Fig. 5C)
GEO dataset GSE102092	P7	Ref [22]	SRR5884807	ATAC-seq (Fig. 5C)
GEO dataset GSE102092	P10	Ref [22]	SRR5884808	ATAC-seq (Fig. 5C)
GEO dataset GSE102092	P14	Ref [22]	SRR5884810	ATAC-seq (Fig. 5C)
GEO dataset GSE102092	P21	Ref [22]	SRR5884811	ATAC-seq (Fig. 5C)
GEO dataset GSE83312	Rod	Ref [23]	SRR3662499	ATAC-seq (Fig. 5C)
GEO dataset GSE83313	Green Cone	Ref [23]	SRR3662503	ATAC-seq (Fig. 5C)
GEO dataset GSE83314	Blue Cone	Ref [23]	SRR3662509	ATAC-seq (Fig. 5C)
ENCODE dataset	Frontal Cortex	Ref [24]	ENCFF018VSA.bam	DNAse footprinting (Fig. 5C)

Supplementary Table 2: Key Resources

Reagent Type	Reagent	Source or reference	Identifier	Additional information
GEO dataset GSE99287	Retina- Macula 1	Ref [25]	SRR5601846	Human ATAC-seq (Fig. 5E)
GEO dataset GSE99287	Retina- Macula 2	Ref [25]	SRR5601851	Human ATAC-seq (Fig. 5E)
GEO dataset GSE99287	Retina- Periphery 1	Ref [25]	SRR5601847	Human ATAC-seq (Fig. 5E)
GEO dataset GSE99287	Retina- Periphery 2	Ref [25]	SRR5601850	Human ATAC-seq (Fig. 5E)
GEO dataset GSE101986	E12.1	Ref [26]	SRR5877174	Mouse whole retina RNA-seq (Fig. 6A)
GEO dataset GSE101986	E12.2	Ref [26]	SRR5877175	Mouse whole retina RNA-seq (Fig. 6C)
GEO dataset GSE101986	E14.1	Ref [26]	SRR5877176	Mouse whole retina RNA-seq (Fig. 6C)
GEO dataset GSE101986	E14.2	Ref [26]	SRR5877177	Mouse whole retina RNA-seq (Fig. 6C)
GEO dataset GSE101986	E16.1	Ref [26]	SRR5877178	Mouse whole retina RNA-seq (Fig. 6C)
GEO dataset GSE101986	E16.2	Ref [26]	SRR5877179	Mouse whole retina RNA-seq (Fig. 6C)
GEO dataset GSE101986	P0.1	Ref [26]	SRR5877180	Mouse whole retina RNA-seq (Fig. 6C)
GEO dataset GSE101986	P0.2	Ref [26]	SRR5877181	Mouse whole retina RNA-seq (Fig. 6C)
GEO dataset GSE101986	P2.1	Ref [26]	SRR5877182	Mouse whole retina RNA-seq (Fig. 6C)
GEO dataset GSE101986	P2.2	Ref [26]	SRR5877183	Mouse whole retina RNA-seq (Fig. 6C)
GEO dataset GSE101986	P4.1	Ref [26]	SRR5877184	Mouse whole retina RNA-seq (Fig. 6C)
GEO dataset GSE101986	P4.2	Ref [26]	SRR5877185	Mouse whole retina RNA-seq (Fig. 6C)
GEO dataset GSE101986	P6.1	Ref [26]	SRR5877186	Mouse whole retina RNA-seq (Fig. 6C)
GEO dataset GSE101986	P6.2	Ref [26]	SRR5877187	Mouse whole retina RNA-seq (Fig. 6C)
GEO dataset GSE101986	P10.1	Ref [26]	SRR5877188	Mouse whole retina RNA-seq (Fig. 6C)
GEO dataset GSE101986	P10.2	Ref [26]	SRR5877189	Mouse whole retina RNA-seq (Fig. 6C)

Supplementary Table 2: Key Resources

Reagent Type	Reagent	Source or reference	Identifier	Additional information
GEO dataset GSE101986	P14.1	Ref [26]	SRR5877190	Mouse whole retina RNA-seq (Fig. 6C)
GEO dataset GSE101986	P14.2	Ref [26]	SRR5877191	Mouse whole retina RNA-seq (Fig. 6C)
GEO dataset GSE101986	P21.1	Ref [26]	SRR5877192	Mouse whole retina RNA-seq (Fig. 6C)
GEO dataset GSE101986	P21.2	Ref [26]	SRR5877193	Mouse whole retina RNA-seq (Fig. 6C)
GEO dataset GSE101986	P28.1	Ref [26]	SRR5877194	Mouse whole retina RNA-seq (Fig. 6C)
GEO dataset GSE101986	P28.2	Ref [26]	SRR5877195	Mouse whole retina RNA-seq (Fig. 6C)
GEO dataset GSE94437	11-1516 Peripheral Retina	Ref [27]	SRR5225761	Human retina RNA-seq (Fig. 6D)
GEO dataset GSE94437	11-1556 Peripheral Retina	Ref [27]	SRR5225765	Human retina RNA-seq (Fig. 6D)
GEO dataset GSE94437	11-1614 Peripheral Retina	Ref [27]	SRR5225769	Human retina RNA-seq (Fig. 6D)
GEO dataset GSE94437	11-1624 Peripheral Retina	Ref [27]	SRR5225773	Human retina RNA-seq (Fig. 6D)
GEO dataset GSE94437	11-1648 Peripheral Retina	Ref [27]	SRR5225777	Human retina RNA-seq (Fig. 6D)
GEO dataset GSE94437	11-1833 Peripheral Retina	Ref [27]	SRR5225781	Human retina RNA-seq (Fig. 6D)
GEO dataset GSE94437	11-1875 Peripheral Retina	Ref [27]	SRR5225785	Human retina RNA-seq (Fig. 6D)
GEO dataset GSE94437	11-2043 Peripheral Retina	Ref [27]	SRR5225789	Human retina RNA-seq (Fig. 6D)
GEO dataset GSE94437	11-1516 Macular Retina	Ref [27]	SRR5225763	Human retina RNA-seq (Fig. 6D)
GEO dataset GSE94437	11-1556 Macular Retina	Ref [27]	SRR5225767	Human retina RNA-seq (Fig. 6D)
GEO dataset GSE94437	11-1614 Macular Retina	Ref [27]	SRR5225771	Human retina RNA-seq (Fig. 6D)
GEO dataset GSE94437	11-1624 Macular Retina	Ref [27]	SRR5225775	Human retina RNA-seq (Fig. 6D)
GEO dataset GSE94437	11-1648 Macular Retina	Ref [27]	SRR5225779	Human retina RNA-seq (Fig. 6D)

Supplementary Table 2: Key Resources

Reagent Type	Reagent	Source or reference	Identifier	Additional information
GEO dataset GSE94437	11-1833 Macular Retina	Ref [27]	SRR5225783	Human retina RNA-seq (Fig. 6D)
GEO dataset GSE94437	11-1875 Macular Retina	Ref [27]	SRR5225787	Human retina RNA-seq (Fig. 6D)
GEO dataset GSE94437	11-2043 Macular Retina	Ref [27]	SRR5225791	Human retina RNA-seq (Fig. 6D)
GEO dataset GSE79416	Cortex_CC1	Ref [28]	SRR3269772	Bulk RNA-seq
GEO dataset GSE79416	Cortex_CC2	Ref [28]	SRR3269773	Bulk RNA-seq
GEO dataset GSE79416	Cortex_CC3	Ref [28]	SRR3269774	Bulk RNA-seq
GEO dataset GSE101544	zf_retina_1	GSE101544	SRR5833542	Bulk RNA-seq
GEO dataset GSE101544	zf_retina_2	GSE101544	SRR5833543	Bulk RNA-seq
GEO dataset GSE59911	Bovine_rep1	GSE59911	SRR1532566	Bulk RNA-seq
GEO dataset GSE59911	Bovine_rep2	GSE59911	SRR1532567	Bulk RNA-seq
GEO dataset GSE59911	Bovine_rep3	GSE59911	SRR1532568	Bulk RNA-seq
GEO dataset GSE84932	rat_rep1	GSE84932	SRR3957262	Bulk RNA-seq
GEO dataset GSE84932	rat_rep2	GSE84932	SRR3957263	Bulk RNA-seq
DDBJ SRA dataset DRA002410	Sham1	Ref [29]	DRX019832	Adult mouse retina CAGE RNA-seq
DDBJ SRA dataset DRA002410	Sham2	Ref [29]	DRX019833	Adult mouse retina CAGE RNA-seq
DDBJ SRA dataset DRA002410	Sham3	Ref [29]	DRX019834	Adult mouse retina CAGE RNA-seq
CRISPR gRNA	Crb1 guide 5’4		GAATAAGTACCCGTTCCTTG	5' guide for making Crb1 AB and B mouse
CRISPR gRNA	Crb1 guide 3’2		AAAGCGATTAGGTGATGCCC	3' guide for making Crb1 B mouse
CRISPR gRNA	Crb1 guide 3’4		TGTCCGAACACGTCAACCCC	3' guide for making Crb1 AB mouse

Supplementary Table 2: Key Resources

Reagent Type	Reagent	Source or reference	Identifier	Additional information
Primer	MegF11 1.1F	IDT	GCTTGCTCACTCGTT CTCAGT	RT-PCR primer
Primer	Megf11_2.1R	IDT	AGCTCTCTCCTTCCA AACCC	RT-PCR primer
Primer	Megf11_alt23_R	IDT	ACCCACAAGCGTTTG CTAAG	RT-PCR primer
Primer	Crb1 delB F1	IDT	CAGTATCCCAGGAGC ATTCC	genotyping primer
Primer	Crb1 delB F2	IDT	TTTTTCAGTGTGCCA GGAAGT	genotyping primer
Primer	Crb1 delB R	IDT	AAGACTTTCCGAAGC CATGA	genotyping primer
Primer	Crb1 delAB F	IDT	CAAGACACCCAGGA CCAAGT	genotyping primer
Primer	Crb1 delAB F2	IDT	CTTCCCTCTTTGGAC ATTGC	genotyping primer
Primer	Crb1 delAB R	IDT	AACTTGGGAGAGCCT GGAGT	genotyping primer
Primer	Crb1_5Fq.seq	IDT	GCCTCGGGCTATGTG TGTAT	qPCR primer
Primer	Crb1_5cFq.seq	IDT	AAACGGTTCCTGTCG ACCTA	qPCR primer
Primer	Crb1_6Rq.seq	IDT	ggcaagggtgcagtaaaca t	qPCR primer
Primer	Crb1_11Fq.seq	IDT	tgcatcaatggaggactgtg	qPCR primer
Primer	Crb1_11UTR_Rq.se q	IDT	tcatgcgcagtacgaggtag	qPCR primer
Primer	Crb1_12Rq.seq	IDT	TGAAGAACAGGGCC AAAGTT	qPCR primer
Primer	Crb1_6Fq.seq	IDT	AGAGGACGCTGCAT CAACTT	qPCR primer
Primer	Crb1_7Rq.seq	IDT	TCATCTTGGCCAAAT CTTCC	qPCR primer
Primer	Crb1_8Fq.seq	IDT	GCTCCCTCAAGGGTT TGAAT	qPCR primer
Primer	Crb1_9Rq.seq	IDT	CCATCAGGTGCAGC GTATAA	qPCR primer
BaseScope Probe	BA-Mm-Megf11-E14E17	Advanced Cell Diagnostics	720881	bp 1999-2304 (NM_001134399.1)
BaseScope Probe	BA-Mm-Megf11-E16bE17	Advanced Cell Diagnostics	720891	bp 2210-2247 (XM_006510984.3)

Supplementary Table 2: Key Resources

Reagent Type	Reagent	Source or reference	Identifier	Additional information
BaseScope Probe	BA-Mm-Megf11-E16E17	Advanced Cell Diagnostics	720901	bp 2264-2302 (NM_001134399.1)
BaseScope Probe	BA-Mm-Megf11-E19E23	Advanced Cell Diagnostics	720911	bp 2699-2744 (XM_006510987.3)
BaseScope Probe	BA-Mm-Megf11-E20E23	Advanced Cell Diagnostics	720921	bp 2822-2864 (XM_006510992.3)
BaseScope Probe	BA-Mm-Megf11-E22E23	Advanced Cell Diagnostics	720931	bp 3050-3086 (NM_001134399.1)
BaseScope Probe	BA-Mm-Megf11-E23E23alt	Advanced Cell Diagnostics	720941	bp 3030-3070 (NM_172522.4)
BaseScope Probe	BA-Mm-Megf11-E23I23	Advanced Cell Diagnostics	720951	bp 64700141-64700189 (NC_000075.6)
BaseScope Probe	BA-Mm-Megf11-E24E25	Advanced Cell Diagnostics	720961	bp 3329-3370 (NM_001134399.1)
BaseScope Probe	BA-Mm-Megf11-E24I24	Advanced Cell Diagnostics	720971	bp 3024-3072 (XM_017313269.1)
BaseScope Probe	BA-Mm-Megf11-E2E3	Advanced Cell Diagnostics	720981	bp 273-314 (NM_001134399.1)
BaseScope Probe	BA-Mm-Crb1-004-E5CE6	Advanced Cell Diagnostics	704351	CACAAGGTTTTCACATTTTAATGGCAGTGCTCATAGGAATTCACTGTG
BaseScope Probe	BA-Mm-Crb1-E1E2	Advanced Cell Diagnostics	704341	ACCTCAGCTCCTCACTGCTCATCTGCATAAAGAATTCATTTTGCA

SUPPLEMENTAL REFERENCES

1. Sokolov, M. *et al.* Phosducin Facilitates Light-driven Transducin Translocation in Rod Photoreceptors. *J. Biol. Chem.* **279**, 19149–19156 (2004).
2. Schindelin, J. *et al.* Fiji: an open-source platform for biological-image analysis. *Nat. Methods* **9**, 676–82 (2012).
3. Trapnell, C. *et al.* Differential gene and transcript expression analysis of RNA-seq experiments with TopHat and Cufflinks. *Nat. Protoc.* **7**, 562–578 (2012).
4. Pertea, M., Kim, D., Pertea, G. M., Leek, J. T. & Salzberg, S. L. Transcript-level expression analysis of RNA-seq experiments with HISAT, StringTie and Ballgown. *Nat. Protoc.* **11**, 1650–1667 (2016).
5. Kim, D., Langmead, B. & Salzberg, S. L. HISAT: A fast spliced aligner with low memory requirements. *Nat. Methods* **12**, 357–360 (2015).
6. Tardaguila, M. *et al.* SQANTI: extensive characterization of long-read transcript sequences for quality control in full-length transcriptome identification and quantification. *Genome Res.* **28**, 396–411 (2018).
7. Robinson, J. T. *et al.* Integrative genomics viewer. *Nat. Biotechnol.* **29**, 24–26 (2011).
8. Dobin, A. *et al.* STAR: Ultrafast universal RNA-seq aligner. *Bioinformatics* **29**, 15–21 (2013).
9. Hahne, F. & Ivanek, R. Visualizing genomic data using Gviz and bioconductor. in *Methods in Molecular Biology* **1418**, 335–351 (2016).
10. Letunic, I. & Bork, P. 20 years of the SMART protein domain annotation resource. *Nucleic Acids Res.* **46**, D493–D496 (2018).
11. Selivanov, D. Text2vec. (2016). Available at: http://text2vec.org/.
12. Wilkins, D. Treemapify. (2020). Available at: https://github.com/wilkox/treemapify.
13. Conway, J. & Gehlenborg, N. UpSetR. (2018). Available at: https://github.com/hms-dbmi/UpSetR.
14. Wu, T. D. & Watanabe, C. K. GMAP: a genomic mapping and alignment program for mRNA and EST sequences. *Bioinformatics* **21**, 1859–1875 (2005).
15. Wickham, H. *et al.* Welcome to the Tidyverse. *J. Open Source Softw.* **4**, 1686 (2019).
16. Wickham, H. Reshaping data with the reshape package. *J. Stat. Softw.* **21**, 1–20 (2007).
17. Galili, T. dendextend. (2019). Available at: https://github.com/talgalili/dendextend/.
18. Krijthe, J. Rtsne. (2020). Available at: https://github.com/jkrijthe/Rtsne.
19. Oksanen, J. *et al.* vegan: Community ecology package. (2020). Available at: https://github.com/vegandevs/vegan.
20. Cochran, K., Ray, T. A. & Kay, J. N. IsoPops: R package for analysis of isoform-diverse transcript datasets. (2019). Available at: https://github.com/kellycochran/IsoPops.
21. Kim, J.-W. *et al.* Recruitment of Rod Photoreceptors from Short-Wavelength-Sensitive Cones during the Evolution of Nocturnal Vision in Mammals. *Dev. Cell* **37**, 520–32 (2016).
22. Aldiri, I. *et al.* The Dynamic Epigenetic Landscape of the Retina During Development,

Reprogramming, and Tumorigenesis. *Neuron* **94**, 550-568.e10 (2017).

23. Hughes, A. E. O., Enright, J. M., Myers, C. A., Shen, S. Q. & Corbo, J. C. Cell Type-Specific Epigenomic Analysis Reveals a Uniquely Closed Chromatin Architecture in Mouse Rod Photoreceptors. *Sci. Rep.* **7**, 43184 (2017).
24. Davis, C. A. *et al.* The Encyclopedia of DNA elements (ENCODE): data portal update. *Nucleic Acids Res.* **46**, D794–D801 (2018).
25. Wang, J. *et al.* ATAC-Seq analysis reveals a widespread decrease of chromatin accessibility in age-related macular degeneration. *Nat. Commun.* **9**, 1364 (2018).
26. Hoshino, A. *et al.* Molecular Anatomy of the Developing Human Retina. *Dev. Cell* **43**, 763-779.e4 (2017).
27. Li, M. *et al.* Comprehensive analysis of gene expression in human retina and supporting tissues. *Hum. Mol. Genet.* **23**, 4001–4014 (2014).
28. Peng, J. *et al.* High-Throughput Sequencing and Co-Expression Network Analysis of lncRNAs and mRNAs in Early Brain Injury Following Experimental Subarachnoid Haemorrhage. *Sci. Rep.* **7**, 46577 (2017).
29. Yasuda, M. *et al.* Retinal transcriptome profiling at transcription start sites: a cap analysis of gene expression early after axonal injury. *BMC Genomics* **15**, 982 (2014).
