# Supplementary material for: Comprehensive identification of mRNA isoforms reveals the diversity of neural cell-surface molecules with roles in retinal development and disease
Source: Nat Commun. 2020 Jul 3;11:3328. doi: 10.1038/s41467-020-17009-7 (PMC7335077; doi:10.1038/s41467-020-17009-7)
Supplement: Supplementary file 3 — Reporting Summary [file 41467_2020_17009_MOESM3_ESM.pdf]

nature research

Corresponding author(s): Jeremy Kay

Last updated by author(s): May 12, 2020

Reporting Summary

Nature Research wishes to improve the reproducibility of the work that we publish. This form provides structure for consistency and transparency in reporting. For further information on Nature Research policies, see Authors & Referees and the Editorial Policy Checklist.

Statistics

For all statistical analyses, confirm that the following items are present in the figure legend, table legend, main text, or Methods section.

n/a	Confirmed	
☐	☒	The exact sample size (*n*) for each experimental group/condition, given as a discrete number and unit of measurement
☐	☒	A statement on whether measurements were taken from distinct samples or whether the same sample was measured repeatedly
☐	☒	The statistical test(s) used AND whether they are one- or two-sided<br>*Only common tests should be described solely by name; describe more complex techniques in the Methods section.*
☐	☒	A description of all covariates tested
☐	☒	A description of any assumptions or corrections, such as tests of normality and adjustment for multiple comparisons
☐	☒	A full description of the statistical parameters including central tendency (e.g. means) or other basic estimates (e.g. regression coefficient) AND variation (e.g. standard deviation) or associated estimates of uncertainty (e.g. confidence intervals)
☐	☒	For null hypothesis testing, the test statistic (e.g. *F*, *t*, *r*) with confidence intervals, effect sizes, degrees of freedom and *P* value noted<br>*Give P values as exact values whenever suitable.*
☒	☐	For Bayesian analysis, information on the choice of priors and Markov chain Monte Carlo settings
☒	☐	For hierarchical and complex designs, identification of the appropriate level for tests and full reporting of outcomes
☐	☒	Estimates of effect sizes (e.g. Cohen's *d*, Pearson's *r*), indicating how they were calculated

Our web collection on statistics for biologists contains articles on many of the points above.

Software and code

Policy information about availability of computer code

Data collection

Olympus CellSense software (version 1.117) and Nikon Elements (version 5.02.00) were used for acquisition of photomicrographs. MassLinx (version 4.1) was used for data acquisition for mass spectrometry and HPLC chromatography. ImageStudio (version 4.0) was used for acquisition of Western Blot images.

Data analysis

We wrote for this project a R package, IsoPops, that is used for clustering of raw reads into isoforms, as well as for downstream analysis of isoform features. It is available under a GNU General Public License v3.0 (https://kellycochran.github.io/IsoPops/index.html).

To generate the entire isoform catalog, the complete dataset (all timepoints, retina and cortex) was analyzed using the cluster function of PacBio Iso-Seq (version 3), with default parameters. Clustering of PacBio reads into isoforms was performed using ToFU (https://github.com/Magdoll/cDNA_Cupcake), which consists of two parts: 1) Isoform-level clustering algorithm ICE (Iterative Clustering for Error Correction), used to generate consensus isoforms; and 2) Quiver, used to polish consensus isoforms. All of these components (ToFU, ICE, and Quiver) were included in Iso-Seq version 3. Finally, additional filtering of putative spurious isoforms was performed with our IsoPops R package. IsoPops was also used for hierarchical clustering/PCA of isoform sequences and for many of the other data visualizations shown for our PacBio dataset.

Isoforms were aligned to the mouse genome mm10 using GMAP (version 1.3.3b) with default values of alignment accuracy (0.85) and coverage (0.99). Sqanti version 1.2 (https://bitbucket.org/ConesaLab/sqanti/src/master/) was used for ORF prediction and genomic correction of PacBio isoforms, as well as for matching of lrCaptureSeq isoforms to other databases. Sashimi plots were generated using Gviz (version 1.24.0). To produce the reference peptide library for proteomics experiments, amino acid sequences were trypsinized in silico using the python program trypsin (no version number given; https://github.com/yafeng/trypsin) with default settings

For analysis of short-read RNA-Seq data: RNA-seq fastq files were mapped with Hisat2 (version 2.1.0). and quantified with Cufflinks (version 2.2.1). or StringTie (version 1.3.3b). Computational prediction of isoforms was performed on the RNA-seq data set GSE101986 and GSE79416 using Cufflinks (version 2.2.1) or Stringtie (version 1.3.3b) without a reference assembly. Resulting assemblies were merged using Cuffmerge (a component of Cufflinks) to create the final reference assembly. Junction coverage of PacBio isoforms by RNA-

October 2018

seq data was assessed using Sqanti software. The junction input file for Sqanti was generated using STAR (STAR_2.6.0a). CAGE RNA-seq data were aligned to the genome (mm10) using Hisat2. Read coverage at exon 1 of the lrCaptureSeq isoforms was determined using BedTools (version 2.29.2). CAGE data coverage across normalized isoform lengths was performed using Qualimap (version 2.2.1). Single-cell RNA-seq data were analyzed using CellRanger (version 3.0) and visualized using Monocle (version 3.0).

Chromatin accessibility: Reads were trimmed using fastqc (version 0.11.3) and trim galore (version 0.4.1) and mapped to either the mm9 or hg19 genomes using bowtie2 (version 2.2.5). Aligned bam files were filtered for quality (>Q30) and mitochondrial and blacklisted regions were removed. Files were converted to bigwigs using deeptools (version 3.1.0) and visualized in IGV (version 2.4.16).

The Shannon index was calculated with the R package Vegan (https://github.com/vegandevs/vegan).

Cell counting and other analysis of histological/ultrastructural images was performed using Fiji (version 1.52n). Figure layout was performed in Adobe Photoshop and Illustrator (creative cloud version 2019).

Alignment of DNA and protein sequence, including ClustalW analysis, was performed with DNASTAR Lasergene (version 10).

Proteomics: To perform peptide assignment to the ion features, ProteinLynx Global Server version 2.5.1 (Waters) was used to generate searchable files that were submitted to the IdentityE search engine incorporated into Progenesis QI for Proteomics (version 4.1). Post-processing, including false discovery rate analysis, was performed using Protein and Peptide Prophet software incorporated into the Scaffold package (version 4.4).

Statistical analysis was performed in Prism software (version 8.3.1).

Chemical structures were illustrated using ChemDraw (version 18.2).

For manuscripts utilizing custom algorithms or software that are central to the research but not yet described in published literature, software must be made available to editors/reviewers. We strongly encourage code deposition in a community repository (e.g. GitHub). See the Nature Research guidelines for submitting code & software for further information.

Data

Policy information about availability of data

All manuscripts must include a data availability statement. This statement should provide the following information, where applicable:

- Accession codes, unique identifiers, or web links for publicly available datasets
- A list of figures that have associated raw data
- A description of any restrictions on data availability

Long-read sequencing data generated in this study have been deposited in the NCBI BioProject repository (accession number PRJNA547800). The Supplementary Data 1 and Supplementary Data 2 files specify the sequence, genomic location, and read number for all isoforms within the lrCaptureSeq dataset.

Mass spectrometry proteomics data generated in this study have been deposited at the ProteomeXchange Consortium via the PRIDE partner repository with the dataset identifier PXD017290 (DOI: 10.6019/PXD017290).

Crb1 isoform cDNA sequences described in this study have been deposited at Genbank with the following accession numbers: MT470365 [https://www.ncbi.nlm.nih.gov/nuccore/MT470365] (human CRB1-A); MT470366 [https://www.ncbi.nlm.nih.gov/nuccore/MT470366] (human CRB1-B); MT470367 [https://www.ncbi.nlm.nih.gov/nuccore/MT470367] (human CRB1-C); MT470368 [https://www.ncbi.nlm.nih.gov/nuccore/MT470368] (mouse Crb1-A); MT470369 [https://www.ncbi.nlm.nih.gov/nuccore/MT470369] (mouse Crb1-B); MT470370 [https://www.ncbi.nlm.nih.gov/nuccore/MT470365] (mouse Crb1-C); and MT470371 [https://www.ncbi.nlm.nih.gov/nuccore/MT470365] (mouse Crb1-A2).

CAGE-seq data used in this study were obtained from the DDBJ sequence read archive (DRA) with accession numbers DRX019832 [https://ddbj.nig.ac.jp/DRASearch/experiment?acc=DRX019832], DRX019833 [https://ddbj.nig.ac.jp/DRASearch/experiment?acc=DRX019833], and DRX019834 [https://ddbj.nig.ac.jp/DRASearch/experiment?acc=DRX019834].

ATAC-seq datasets used in this study were obtained from the NCBI Gene Expression Omnibus. The accession numbers for these data were as follows: 1) GSE102092 [https://www.ncbi.nlm.nih.gov/geo/query/acc.cgi?acc=GSE102092]; 2) GSE83312 [https://www.ncbi.nlm.nih.gov/geo/query/acc.cgi?acc=GSE83312]; 3) GSE99287 [https://www.ncbi.nlm.nih.gov/geo/query/acc.cgi?acc=GSE99287].

Bulk RNA-seq datasets used in this study were obtained from NCBI GEO with the following accession numbers: 1) GSE101986 [https://www.ncbi.nlm.nih.gov/geo/query/acc.cgi?acc=GSE101986]; 2) GSE94437 [https://www.ncbi.nlm.nih.gov/geo/query/acc.cgi?acc= GSE94437]; 3) GSE74660 [https://www.ncbi.nlm.nih.gov/geo/query/acc.cgi?acc=GSE74660]; 4) GSE101544 [https://www.ncbi.nlm.nih.gov/geo/query/acc.cgi?acc=GSE101544]; 5) GSE59911 [https://www.ncbi.nlm.nih.gov/geo/query/acc.cgi?acc=GSE59911]; 6) GSE84932 [https://www.ncbi.nlm.nih.gov/geo/query/acc.cgi?acc=GSE84932]; 7) GSE79416 [https://www.ncbi.nlm.nih.gov/geo/query/acc.cgi?acc=GSE79416]; 8) GSE74660 [https://www.ncbi.nlm.nih.gov/geo/query/acc.cgi?acc=GSE74660]. The scRNA-seq dataset used in this study is available from NCBI GEO with accession number GSE118614 [https://www.ncbi.nlm.nih.gov/geo/query/acc.cgi?acc=GSE118614].

For proteomics experiments, the entire UniProtKb database [https://www.uniprot.org/help/uniprotkb] was used as a reference database.

The source data underlying graphs in Fig. 6C,D; Fig. 8C, Fig. 9J; Fig. 10C,D; Supplementary Fig. 5A,B; Supplementary Fig. 6B; and Supplementary Fig. 7C,F are provided in a Source Data file. Also see the Source Data file for full gel images related to Fig. 7C,D; Fig 8D; Supplementary Fig. 5C; and Supplementary Fig. 7B.

Field-specific reporting

Please select the one below that is the best fit for your research. If you are not sure, read the appropriate sections before making your selection.

☒ Life sciences ☐ Behavioural & social sciences ☐ Ecological, evolutionary & environmental sciences

For a reference copy of the document with all sections, see nature.com/documents/nr-reporting-summary-flat.pdf

Life sciences study design

All studies must disclose on these points even when the disclosure is negative.

Sample size

Pre-calculation of sample size was not performed. Sample sizes for genetic experiments involving mutant mice were largely determined by the number of breeders available and the genotypes of the offspring. It was not anticipated that large sample sizes would be needed based on the size of the hypothesized effects. For surveys of gene or protein expression (e.g. PacBio sequencing, proteomics), all mice from an individual litter (typically n=5-6 mice) were pooled so as to smooth any individual expression differences across the broad reference datasets. For qualitative histology or Western blot experiments in which statistics were not performed, we ensured that expression patterns were consistent across independent experiments or animals (see "Replication" below). This approach ensured that observed staining patterns were not idiosyncratic to particular animals or litters.

Data exclusions

Low abundance isoforms (those contributing to bottom 5% of total cDNA expression) were excluded from our analysis of isoform numbers. This was done out of an abundance of caution to ensure that we were not reporting spurious isoforms as real ones. The 5% cutoff was not pre-established, but was chosen based on the fact that the bottom 5% of isoforms did not contribute significantly to the expressed isoform diversity. Because 5% is a somewhat arbitrary cutoff we also have shown our data using different cutoffs so that readers can judge for themselves the impact of cutoff level (see Supplementary Figures S2D; S3A,B).

Replication

The lrCaptureSeq experiments were performed once for each condition/age (4 mouse retina timepoints; 1 mouse brain timepoint; 1 human retina condition). Mouse experiments used one C57Bl6/J animal for each condition. Human experiments used tissue from a single donor (male, age 59). Each proteomics strategy (i.e. cell surface biotinylation and trypsin ectodomain release) was performed once, although the biotinylation and pull-down conditions were worked out in pilot experiments. The gel shown in Fig. 3f is the same one used for the proteomics experiment and was representative of the pilot experiments using similar conditions. For each proteomics strategy, P14 mouse retinal tissue was pooled from multiple littermates. Even though the large-scale sequencing and mass spectrometry experiments were performed only once, we replicated key results using different experimental approaches, such as corroboration with independent short-read datasets (Fig. 6C,D; Supplementary Fig. 2B); CAGE-seq datasets (Supplementary Fig. 1D); and qPCR (Supplementary Fig. 5B,C).

PacBio sequencing was performed on two different retinal Megf11 RT-PCR reactions as shown in Supplementary Fig. 4. Together with lrCaptureSeq, therefore, we had 3 independent PacBio datasets for the Megf11 gene. RT-PCR gel images shown in Supplementary Fig. 4A are representative of many such reactions that were performed. These images are also representative of the reactions that were used for PacBio sequencing.

Mouse mutant phenotypes reported in Fig. 9, Fig. 10, and Supplementary Fig. 7B-F have been observed in multiple animals and multiple litters from different founder strains of the mutant alleles. Since the phenotypes were consistent, these groups were pooled for the analysis and reported as a single experiment. Photomicrographs in these figures are representative of the phenotypes, or the range of phenotypes, observed across animals of a given genotype.

Western blots of CRB1-B expression in delB mutants (Fig. 7C, D) are representative examples of results obtained from 3 independent experiments (i.e. 3 biological replicates). The CRB1-B blot from null mutant (Supplementary Fig. 7B) is a representative example of an experiment that was repeated twice (2 biological replicates). The serial section Western blotting experiment was repeated three times (3 biological replicates); images shown in Fig. 8D are from a single experiment. They are representative examples of the results obtained each time.

Images shown in Fig. 4E and Fig. 8C are representative of BaseScope staining that was repeated at least three times on retinal tissue from separate animals. Images of transfected K562 cells (Supplementary Fig. 6C) are representative of two independently transfected tissue culture coverslips, which were imaged in parallel. The Crb1 qPCR experiment (Supplementary Fig. 5B) was performed once, although results were consistent with smaller pilot experiments in which primers and conditions were being tested. The Crb1 RT-PCR gel (Supplementary Fig. 5C) was run twice on different RNA samples with identical results.

Randomization

Mice were allocated into groups based on their genotype. Isoforms were allocated into groups based on the gene that encodes them.

Blinding

The only experiments with multiple experimental groups were the ones involving Crb1 mutants. Histological quantification was performed blind to genotype. QPCR samples were quantified automatically so user bias could not have contributed to the results. Quantification of publicly available sequencing datasets was likewise performed in an automated fashion so that it would not be influenced by user bias.

Reporting for specific materials, systems and methods

We require information from authors about some types of materials, experimental systems and methods used in many studies. Here, indicate whether each material, system or method listed is relevant to your study. If you are not sure if a list item applies to your research, read the appropriate section before selecting a response.

Materials & experimental systems

n/a	Involved in the study
☐	☒ Antibodies
☐	☒ Eukaryotic cell lines
☒	☐ Palaeontology
☐	☒ Animals and other organisms
☐	☒ Human research participants
☒	☐ Clinical data

Methods

n/a	Involved in the study
☒	☐ ChIP-seq
☒	☐ Flow cytometry
☒	☐ MRI-based neuroimaging

Antibodies

Antibodies used	Donkey Anti-rabbit IgG 1:1000 Jackson ImmunoResearch 711-545-152<br>Rabbit anti-Calbindin 1:5000 Swant CB-38<br>Rabbit anti-CRB1-B: 1:500 this study<br>goat anti-ABCA4 1:1000 Santa Cruz sc21460<br>mouse anti rhodopsin clone 1D4 1:1000 Abcam ab5417<br>mouse anti-GAPDH clone 0411 1:1000 Santa Cruz sc-47724<br>Sheep anti-phosducin 1:5000 Sokolov et al., 2004<br>IRDye 800CW Donkey anti-Rabbit IgG (H + L): 1:1000 Li-Cor Biosciences 925–32213<br>IRDye 680RD Donkey anti-Mouse IgG (H + L): 1:1000 Li-Cor Biosciences 925–68072
Validation	The CRB1-B antibody is validated in this study, using knockout mouse tissue. Calbindin is a well established marker of mouse starburst amacrine cells and horizontal cells. These cells can be identified by morphological features that are clearly evident in Fig. 4E. ABCA4, rhodopsin, GAPDH, and phosducin antibodies recognize bands of expected size in Western blots (Fig. 7 C,D; Fig. 8D). Phosducin and ABCA4 were specifically detected in soluble and membrane fractions,(Fig. 7D) while Rhodopsin was specifically detected in outer segment tissue (Fig. 8D), further validating the specificity of these antibodies. Finally, the ABCA4 and phosducin antibodies were previously validated using knockout mouse tissue in other studies (ABCA4: https://doi.org/10.1073/pnas.1802519115; phosducin: Sokolov et al. (2004) cited in the Supplementary references).

Eukaryotic cell lines

Policy information about cell lines

Cell line source(s)	K562 cells were acquired from ATCC
Authentication	Authentication was performed by ATCC at time of acquisition. No further subsequent authentication was done in our laboratory.
Mycoplasma contamination	Mycoplasma test was performed by ATCC at time of acquisition. The cells tested negative. No further subsequent testing was done in our laboratory.
Commonly misidentified lines (See ICLAC register)	none

Animals and other organisms

Policy information about studies involving animals; ARRIVE guidelines recommended for reporting animal research

Laboratory animals	Mus musculus: C57Bl/6J strain (all experiments except Crb1 mutant studies). Crb1-delB and Crb1-null mutant mouse strains were generated in this study, on a mixed SJL; C57Bl/6J background. Various ages from postnatal day 1 to adulthood were used; the precise ages are noted in each figure and/or figure legend. Animals of both sexes were used for all experiments. The mice were housed under a 12 hr light-dark cycle with ad lib access to food and water. Heat and humidity were maintained within the parameters specified in the National Institute of Health Guide for the Care and Use of Laboratory Animals.
Wild animals	none used
Field-collected samples	none used
Ethics oversight	Mouse experiments in this study were approved by the Duke University Institutional Animal Care and Use Committee (protocols A005-16-01 and A274-18-12). The mice were housed under a 12 hr light-dark cycle with ad lib access to food and water. Heat and humidity were maintained within the parameters specified in the National Institute of Health Guide for the Care and Use of Laboratory Animals. Experimental procedures were also consistent with this Guide.

Note that full information on the approval of the study protocol must also be provided in the manuscript.

Human research participants

Policy information about studies involving human research participants

Population characteristics	One retina from a 59 year old male postmortem donor was used. The donor had no history of retinal disease, which eliminated an important possible source of confounding effect on retinal gene expression.
Recruitment	Voluntary organ donations were the source of the eyes obtained by Miracles in Sight (Winston Salem, NC). The tissues are distributed to Duke research labs by BioSight, a Duke University Shared Resource.<br><br>The goal of the study was to evaluate gene expression in normal adult human retina. It is possible that postmortem donor tissue, which was the only tissue available to us, is not entirely representative of the broad population. This could in theory affect the interpretations of this study. However it is unlikely that our specific donor demographics and/or recruitment strategy had a major effect on our conclusions, as our CRB1 expression results were corroborated by independent RNA-seq datasets published by other laboratories (see Fig. 6).
Ethics oversight	Human donor eyes were obtained from Miracles in Sight (Winston Salem, NC), which were distributed by BioSight (Duke University Shared Resource). Ethical procedures, including procedures for obtaining informed consent from donors, were reviewed and approved by the Duke University Institutional Review Board (protocol #PRO-00050810).

Note that full information on the approval of the study protocol must also be provided in the manuscript.
