# Supplementary material for: Comprehensive identification of mRNA isoforms reveals the diversity of neural cell-surface molecules with roles in retinal development and disease
Source: Nat Commun. 2020 Jul 3;11:3328. doi: 10.1038/s41467-020-17009-7 (PMC7335077; doi:10.1038/s41467-020-17009-7)
Supplement: Supplementary file 4 — Description of Additional Supplementary Files [file 41467_2020_17009_MOESM4_ESM.pdf]

**Description of Additional Supplementary Files**

File Name: Supplementary Data 1
Description: mRNA and ORF isoforms identified in this study. An Excel file with the complete sequence of each isoform detected in this study, along with its genomic location and abundance within our full dataset. The protein sequence of the predicted ORF is also provided. The Excel file has a “Legend” tab which defines all data provided within the table.

File Name: Supplementary Data 2
Description: GFF file for mRNA isoforms identified in this study. The text file “fullfilter.gff.txt” (GFF formatting) is provided as a companion to Supplementary Data 1. It may be used for mapping of the Supplementary Table 1 isoforms within genome browser software.

File Name: Supplementary Data 3
Description: Unannotated peptides identified in proteomics experiment. An Excel file with the sequences of the unannotated peptides detected in our proteomics experiment. Listed peptides are absent from UniProt database typically used for matching to mass spectrometry data. Some peptides are predicted to exist in public databases without any experimental support; these peptides are noted in the table. The Excel file has a “Legend” tab which defines all data provided within the table.
